# The effect of vitamin D on fibroblast growth factor 23: a systematic review and meta-analysis of randomized controlled trials

**DOI:** 10.1038/s41430-020-00725-0

**Published:** 2020-08-27

**Authors:** Armin Zittermann, Heiner K. Berthold, Stefan Pilz

**Affiliations:** 1 Clinic for Thoracic and Cardiovascular Surgery, Herz- und Diabeteszentrum NRW, Ruhr University Bochum, Bad Oeynhausen, 32545 Germany; 2 Department of Internal Medicine and Geriatrics, Bethel Clinic (EvKB), Bielefeld, 33611 Germany; 3 Division of Endocrinology and Diabetology, Department of Internal Medicine, Medical University of Graz, Graz, 8036 Austria

**Keywords:** Biomarkers, Endocrine system and metabolic diseases, Cardiovascular diseases

## Abstract

The phosphaturic hormone fibroblast growth factor 23 (FGF23) is a risk marker of cardiovascular and all-cause mortality. We therefore aimed to synthesize the evidence for the effect of vitamin D administration on circulating FGF23 concentrations. We performed a systematic review and meta-analysis of randomized, placebo-controlled trials (RCTs) in several databases from inception to January 2020. A total of 73 records were identified for full-text review, and 21 articles with 23 studies were included in the final analysis. The selected studies included 1925 participants with 8–156 weeks of follow-up. The weighted mean difference in FGF23 in the vitamin D versus placebo group was +21 pg/ml (95% CI: 13–28 pg/ml; *P* < 0.001) with considerable heterogeneity among studies (*I*^2^ = 99%). The FGF23 increment was higher in patients with end-stage kidney/heart failure than in other individuals (+300 pg/ml [95% CI: 41–558 pg/ml] vs. +20 pg/ml [95% CI: 12–28 pg/ml], *P*_interaction_ = 0.03), and if baseline 25-hydroxyvitamin D concentrations were <50 nmol/l instead of ≥50 nmol/l (+34 pg/ml [95% CI: 18–51 pg/ml] vs. +9 pg/ml [95% CI: 3–14 pg/ml]; *P*_interaction_ = 0.002). Moreover, the FGF23 increment was influenced by vitamin D dose/type (vitamin D dose equivalent ≤ 2000 IU/day: +2 pg/ml [95% CI: 0–3 pg/ml]; vitamin D dose equivalent > 2000 IU/day: +18 pg/ml [95% CI: 6–30 pg/ml]; administration of activated vitamin D: +67 pg/ml [95% CI: 16–117 pg/ml]; *P*_interaction_ = 0.001). Results were not significantly influenced by study duration (*P*_interaction_ = 0.14), age class (*P*_interaction_ = 0.09), or assay provider (*P*_interaction_ = 0.11). In conclusion, this meta-analysis of RCTs demonstrates that vitamin D administration of >2000 IU/d vitamin D or activated vitamin D significantly increased concentrations of the cardiovascular risk marker FGF23, especially in patients with end-stage kidney/heart failure.

## Introduction

Fibroblast growth factor 23 (FGF23) plays a pivotal role in the regulation of mineral metabolism: The hormone has phosphaturic properties, augments renal calcium and sodium reabsorption, and is stimulated by high serum phosphate concentrations to maintain serum phosphate within the normal range [1, 2]. FGF23 also activates the renin-angiotensin-aldosterone system and leads to cardiac left ventricular hypertrophy by a calcineurin- and nuclear factor of activated T cells (NFAT)-mediated process [3, 4]. Although FGF23 is primarily secreted by bone cells [5], cardiomyocytes are also capable of synthesizing this hormone [2].

FGF23 is an important marker of poor clinical outcome. Briefly, in patients with c-terminal FGF23 (cFGF23) values > 78 RU (research units)/ml, the multivariable-adjusted risks of all-cause and cardiovascular mortality were 34% and 28%, respectively, higher than in patients with values < 40 RU/ml [6]. Moreover, a meta-analysis of prospective cohort studies indicates that the risk of all-cause mortality rises gradually and progressively as cFGF23 increases, with a fivefold higher risk at cFGF23 values of 300 RU/ml than of 25 RU/ml [7]. The cause and effect relationship regarding the association between FGF23 and outcome is still elusive, but considering the wide use of vitamin D treatment, along with some concerns regarding potential adverse vitamin D effects at high doses, there exists a need to better characterize the vitamin D effect on FGF23.

From a pathophysiological point of view, vitamin D improves the efficacy of intestinal phosphorus absorption from 60% to 80% [8]. Higher circulating concentrations of 25-hydroxyvitamin D (25[OH]D), which is the hallmark of assessing vitamin D status, are associated with an increase in urinary phosphorus excretion [9]. In line with this, vitamin D supplementation and administration of the hormonal form of vitamin D, 1,25(OH)_2_D_3_ (calcitriol), also increase urinary phosphorus levels and may therefore stimulate FGF23 secretion [10, 11].

The present systematic review and meta-analysis therefore aimed to synthesize the effect of vitamin D administration on circulating FGF23 concentrations.

## Methods

This meta-analysis was planned, conducted, and reported on the basis of a protocol that was developed in accordance with the PRISMA statement [12]. The protocol was registered at the PROSPERO international prospective register of systematic reviews as CRD42020171861.

### Eligibility criteria

To be included, studies had to be a randomized controlled trial (RCT) with a control group receiving a placebo instead of vitamin D (Table 1). Interventions using a cross-over design with a placebo period were also eligible. Measurement of change in FGF23 was a necessary condition for study eligibility, and we excluded RCTs which did not report this change for each study cohort separately. We applied no language or time restrictions, and there were no limitations with regard to patient characteristics or vitamin D dose.

**Table 1:** PICOS criteria for inclusion or exclusion of studies.

Parameters	Inclusion criteria	Exclusion criteria
Population	Human individuals	None
Intervention	Administration of vitamin D or activated vitamin D	Non-oral administration
Comparison	FGF23 change by vitamin D vs. placebo	No reporting of baseline and/or in-study FGF23 values
Outcome	Incremental FGF23	No separate reporting of incremental FGF23 by study group
Study design	Only RCTs or studies with a cross-over design	No placebo group/period

*FGF23* fibroblast growth factor 23, *RCT* randomized controlled trial.

### Search strategy

We performed a systematic literature search for publications up to 31 January 2020 in several databases, such as PubMed, Web of Science, the Cochrane Library for reports, and clinicaltrials.gov. We used the following search terms: [cholecalciferol or ergocalciferol or vitamin D or calcitriol or 1,25-dihydroxyvitamin D or 1α-vitamin D or paricalcitol or activated vitamin D] and [supplementation or administration or use] and [RCT] and [FGF23]. We searched for the keywords in the titles and in the abstract, when available. Titles and abstracts of records identified in the primary search were screened, and all articles deemed potentially eligible for inclusion were retrieved in full-text format. Abstracts and unpublished results were not included. To identify additional papers, the reference lists of included studies and published reviews were also scanned. The search was performed independently by two researchers (AZ and HKB). Disagreements were resolved after debate by consensus.

### Data extraction

We performed data extraction with the use of a protocol designed before we conducted the data searches. The following information was extracted: definition of intervention and control, change in FGF23 in each arm, and important baseline characteristics of the study population (Table 2). In cases in which vitamin D was not administered daily, we calculated the daily dose by dividing the administered dose by its frequency. Moreover, we recorded the method of FGF23 measurement, as well as the measured form of FGF23 (i.e., c-terminal or intact FGF23 [iFGF23]).

**Table 2:** Characteristics of 21 articles on vitamin D/activated vitamin D administration.

	Administration of vitamin D/activated vitamin D	
	Number	Percent
Intervention groups	23	-
Subjects		
Intervention	1025	-
Placebo	900	-
Region of origin		
Europe	11	48
America	8	35
Asia	3	13
Australia	1	4
Age		
<60 years	13	57
≥60 years	9	39
Not specified	1	4
Health status		
Apparently healthy	6	26
End-stage organ failure	6	26
Other patients	11	48
Study duration		
<52 weeks	18	78
≥52 weeks	5	22
Baseline 25(OH)D (nmol/l)		
<50 nmol/l	15	65
≥50 nmol/l	7	30
Not specified	1	4
Baseline 1,25(OH)_2_D (pmol/l)		
<40 pmol/l	2	9
40–70 pmol/l	6	26
>70 pmol/l	10	43
Not specified	5	22
Vitamin D type		
D_2_	3	13
D_3_	15	65
Calcitriol	2	9
Paricalcitol	3	13
Vitamin D dose (D2/D3)		
≤2000 IU/day	5	22
>2000 IU/day	13	57
Activated vitamin D (dose)		
≤0.5 µg/day	2	9
<0.5 µg/day	3	13
Frequency of Intake		
Daily	13	57
Weekly	5	22
Monthly	1	4
Bimonthly	1	4
Other	3	13
Assay provider		
Kainos	13	57
Immutopics	6	26
Other	4	17

### Data synthesis

We assessed mean differences in circulating FGF23 concentrations between intervention and control groups. Data are presented as weighted mean differences of the groups with their 95% confidence interval (CI). We used a more conservative random effects model to consider potential heterogeneity among studies. Information on mean and SD values was extracted from the text or from figures. When differences between baseline and in-study values were reported as median and range, we used the formula by Hozo et al. [13] to estimate mean and SD. Results are given as pg/ml. If FGF23 concentrations were presented as RU/ml, we used a correction factor of 0.5 to convert FGF23 concentrations to pg/ml [14]. Moreover, a correction factor of 7.52 was applied to convert pmol/l into pg/ml. If an article consisted of more than one treatment arm, the number of patients included in the placebo arm was divided by the number of treatments.

The extent of between-study heterogeneity was also assessed by *I*^2^ statistics, thereby classifying 25%, 50%, and 75% as low, moderate, and high degrees of heterogeneity, respectively [15].

### Data analysis

Several subgroup analyses were performed. To understand the degree to which the effect of vitamin D supplementation on FGF23 may be explained by its dose or the vitamin D type administered, we conducted meta-analyses by trials with daily vitamin D doses ≤2000 international units (IU), >2000 IU, and those administering activated vitamin D. To explore the potential for a disease-related effect, meta-analyses were conducted in patients without and with end-stage organ failure (i.e., those with chronic kidney disease stage [CKD] 5 or awaiting a heart transplant). Additional meta-analyses were performed by baseline 25(OH)D concentrations (< 50 nmol/l or ≥ 50 nmol/l), study duration (<52 weeks or ≥52 weeks), age class (<60 years or ≥60 years), and assay provider. We also assessed the effect of achieved 25(OH)D concentrations (<100 nmol/l or ≥100 nmol/l) in studies using native vitamin D or 25(OH)D. To determine whether a statistically significant subgroup difference was detected, the test for subgroup differences from the Revman statistics program (see below) was used. This test tests the difference between the pooled effect estimates for each subgroup. All data for subgroup analyses were available from the original articles.

We conducted sensitivity analyses excluding trials that used vitamin D_2_, calcitriol, paricalcitol, or bolus administration of vitamin D_3_, and where baseline FGF23 differed substantially between the vitamin D and placebo groups.

To investigate whether publication bias might affect the validity of the estimates, we constructed funnel plots of the regression of observed effect sizes against the corresponding SEs, weighted by the inverse of the pooled variance [16]. Study quality was assessed according to a tool provided by the Cochrane Handbook for Systematic Reviews of Interventions [17].

For statistical significance, two-sided *α* was set at *P* < 0.05. All statistical analyses were conducted using RevMan (Review Manager. Version 5.3.: The Nordic Cochrane Centre. The Cochrane Collaboration. Copenhagen, 2014).

## Results

### Included studies

**Fig. 1:**
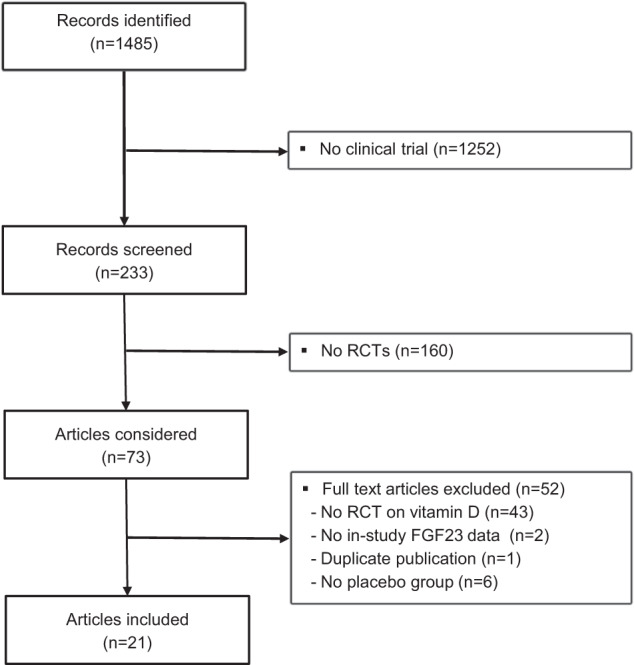
Flowchart of identified and selected studies. Flowchart of selection of studies for inclusion in the meta-analysis.

In total, we identified 1485 abstracts (Fig. 1). We excluded 1252 abstracts because the studies were not clinical trials, leaving 233 records for screening. Of these, we excluded 160 on the basis of screening titles and abstracts because they were not RCTs. Therefore, 73 studies were considered for systematic review by inspecting full-text articles. Of these, we excluded an additional 52 articles because they were not an RCT on vitamin D, no placebo group was included, no in-study FGF23 values were presented, or because of duplicate publication. Thus, we included 21 articles in our systematic review (see Supplementary Table 1). Two articles consisted of 2 studies each, resulting in 23 studies that were finally included in our analysis. Our search did not identify articles of interest for our review in languages other than English. The characteristics of the studies are shown in Table 2. They were published between 2012 and 2019. The trials comprised 1025 study participants in the intervention group and 900 in the control groups. Out of the 23 studies, 17 studies tested native vitamin D (doses equivalent to ≤2000 IU daily in 3 studies and >2000 IU daily in 14 studies, range: 400–8000 IU daily), 1 tested 25(OH)D (dose equivalent 2143 IU/d), 5 tested activated vitamin D (two tested calcitriol [0.21 and 0.5 µg/day] and 3 tested paricalcitol [1–3 µg/day]). Mean baseline 25(OH)D values were <50 nmol/l in 15 studies and ≥50 nmol/l in 8 studies. Of the 23 studies, six were performed in patients with end-stage organ failure (CKD stage 5, *n* = 5; HF stage D, *n* = 1). Five studies analyzed cFGF23 values, whereas 18 studies measured iFGF23 values. Assays were provided by Kainos Laboratories (*n* = 13), Immutopics Inc. (*n* = 6), and others (*n* = 4).

### Synthesis of results

**Fig. 2:**
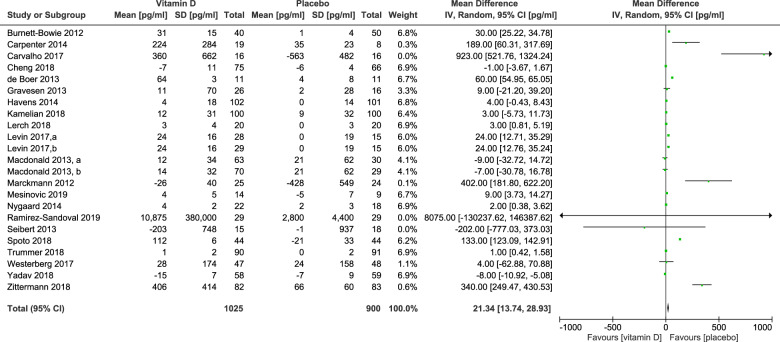
Effect of vitamin D on circulating FGF23 concentrations. Data represent mean differences in circulating FGF23 concentrations between intervention and control groups with 95% confidence interval of individual studies and total effect.

Substitution with vitamin D or activated vitamin D increased circulating FGF23 by a mean of 21 pg/ml (95% CI: 14–29 pg/ml; *P* < 0.001) (Fig. 2). There was an evidence for a significant heterogeneity among studies (*I*^2^ = 99%), confirming the need for a random effect model.

**Fig. 3:**
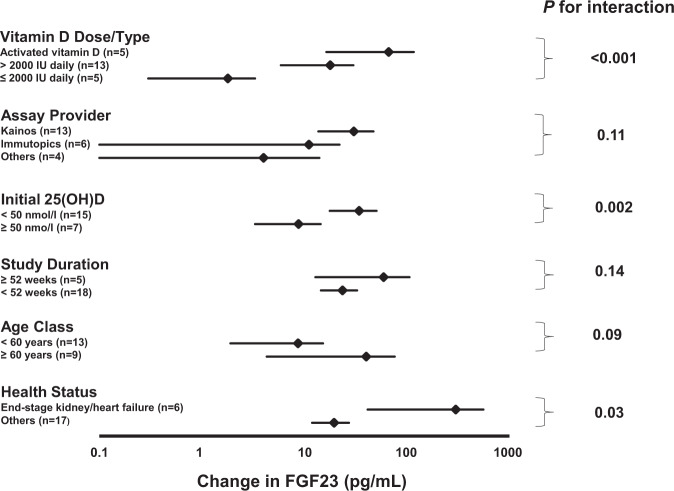
Subgroup analyses of vitamin D on mean differences in circulating FGF23 concentrations between intervention and control groups. Boxes represent the mean values, and error bars indicate 95% confidence intervals of mean differences in FGF23 concentrations in subgroups of randomized trials. Results are given in pg/mL. Numbers indicate number of trials in each subgroup and *P* values refer to differences between subgroups.

To investigate heterogeneity, we therefore evaluated in subgroup analyses the effect of vitamin D dose/type, health status, duration of vitamin D administration, baseline 25(OH)D concentration, age class, and method of measurement (Fig. 3). The FGF23 increment was higher in patients with end-stage kidney/heart failure than in other subjects (+300 pg/ml [95% CI: 41–558 pg/ml] vs. +20 pg/ml [95% CI: 12–27 pg/ml]), and was higher if baseline 25(OH)D concentrations were <50 nmol/l instead of ≥50 nmol/l (+34 pg/ml [95% CI: 18–51 pg/ml] vs. +9 pg/ml [95% CI: 3–14 pg/ml]). Moreover, the FGF23 increment was influenced by vitamin D dose/type (daily vitamin D dose ≤ 2000 IU: +2 pg/ml [95% CI: 0–3 pg/ml]; daily vitamin D dose > 2000 IU:+18 pg/ml [95% CI: 6–30 pg/ml]; administration of activated vitamin D: +67 pg/ml [95% CI: 16–117 pg/ml]). Of the five studies on activated vitamin D, three were performed in patients with CKD stages 3–4. The effect of activated vitamin D on FGF23 was also significantly higher in comparison to the four studies in patients with CKD stages 2–4, who received native vitamin D (+72 pg/ml [95% CI: 19–126 pg/ml] vs. +5 pg/ml [95% CI: −6 to 15 pg/ml] *P* = 0.01). Results were not significantly influenced by study duration (*P*_interaction_ = 0.14), age class (*P*_interaction_ = 0.09), or assay provider (*P*_interaction_ = 0.11). In the 18 studies that used native vitamin D or 25(OH)D (Supplementary Table 2), the effect on FGF23 was significantly higher in studies with achieved circulating 25(OH)D concentrations ≥100 nmol/l than in studies with achieved 25(OH)D <100 nmol/l (65 pg/ml [95% CI: 30–99 pg/ml] vs. 1 pg/ml [95% CI: −1 to 4 pg/ml] *P* < 0.001).

### Sensitivity analysis

Exclusion of one trial, whose baseline FGF23 differed substantially between the vitamin D and placebo group [18], revealed effects of vitamin D consistent with the main analysis (21 pg/ml [95% CI: 14–29 pg/ml; *P* < 0.001]). Sensitivity analysis restricted to trials that used daily vitamin D_3_ supplements was consistent with effects of low-dose vitamin D supplementation (4 pg/ml [95% CI: −1 to 4 pg/ml; *P* = 0.08]).

### Publication bias and study quality

Inspection of the funnel plot of included studies does not exclude the possibility of publication bias (Supplementary Fig. 1). Regarding study quality, there was some risk of bias, especially regarding blinding of participants/personnel and incomplete outcome data presentation (Supplementary Fig. 2).

## Discussion

In this meta-analysis of RCTs, we found that vitamin D administration was associated with a significant increase in FGF23 concentrations. The effect was dose-dependent and influenced by health status, and baseline as well as achieved 25(OH)D concentrations.

The vitamin D-induced mean increase in FGF23 of 21 pg/ml approximates an increase in cFGF23 of 43 RU/ml [14]. Since most assays have set the upper reference value for iFGF23 at 50–100 pg/ml and for cFGF23 at 100 RU/ml, our results indicate a substantial vitamin D effect on circulating FGF23. An increase in cFGF23 of 43 RU/ml has been shown to be associated with a greater cardiovascular and all-cause mortality of approximately roughly 30% [5].

It is, however, also noteworthy that there was a null effect on FGF23 concentrations of daily vitamin D doses ≤2000 IU, whereas the mean increase in FGF23 was 18 pg/ml at daily vitamin D doses >2000 IU. Thus, our results indicate that the officially recommended daily vitamin D dose of 600–800 IU [19] may not affect FGF23 concentrations. This assumption is also in line with our findings that vitamin D supplementation increases FGF23 concentrations only if achieved circulating 25(OH)D concentrations are above 100 nmol/l, but not if achieved 25(OH)D concentrations remain below 100 nmol/l.

The phosphaturic hormone FGF23 is an important negative endocrine regulator of calcitriol synthesis, as well as intestinal phosphorus absorption, and may therefore contribute to the prevention of phosphorus intoxication [20]. In line with the null effect of physiologic oral vitamin D doses on FGF23 concentrations, the effect of supplemental vitamin D on circulating calcitriol is dose-dependent, with a significant lower increase at daily vitamin D doses ≤1000 IU than at doses >1000 IU [21]. Thus, at physiologic oral vitamin D doses the human body may be protected from the need to increase FGF23. The absent/small effect of physiologic amounts of supplemental vitamin D on the cardiovascular risk marker FGF23 in the present meta-analysis also supports earlier large meta-analyses showing no adverse effect of supplemental vitamin D on cardiovascular morbidity and mortality [22, 23].

Administration of activated vitamin D resulted in higher FGF23 concentrations than supplementation with native vitamin D. Activated vitamin D does not need a 1α-hydroxylation. Therefore, in the clinical setting it is often prescribed in patients with diseases that result in limited calcitriol synthesis. In the present meta-analysis, three out of the five studies on activated vitamin D were performed in patients with CKD stages 3–4. Even in comparison to RCTs that used supplements of native vitamin D in patients with a similar health status (CKD stages 2–4), activated vitamin D resulted in a higher increase in FGF23. In CKD patients, administration of activated vitamin D tends to result in a much higher rise in circulating calcitriol than in non-CKD patients [20]. Importantly, in children on dialysis a U-shaped association has been reported between circulating calcitriol concentrations and carotid intima thickness or calcification score [24]. Although multiple studies suggest an association between the use of active vitamin D therapy in patients on dialysis and with CKD and improved survival, there are also many studies indicating important adverse effects of such a treatment [25]. Altogether, our data do not rule out the possibility that in some CKD patients administration of activated vitamin D contributes to the high cardiovascular risk of these patients. In a relatively large recent trial in patients undergoing maintenance hemodialysis [26], the risk of a composite measure of fatal and nonfatal cardiovascular events tended to be higher in patients receiving activated vitamin D versus usual care, especially when the per-protocol set was analyzed. However, it is also noteworthy that in patients with CKD stages 2–4, a well-known group of patients of a relatively high CVD risk [27], the weighted mean increase in FGF23 by native vitamin D supplementation was rather small (+5 pg/ml). Thus, there may be a relatively low risk of CVD deterioration by native vitamin D in this group of patients. Although observational and mechanistic studies suggest some cardiovascular protective actions of vitamin D, RCTs did not clearly document beneficial or adverse effects of vitamin D supplementation on cardiovascular outcomes [28].

In the present meta-analysis, the vitamin D-induced increase in FGF23 was the highest in patients with end-stage organ failure requiring hemodialysis/awaiting heart transplantation. Notably, none of the studies in patients with end-stage organ failure used activated vitamin D, indicating that the severity of kidney/heart failure may be even more important for the vitamin D-induced increase in FGF23 than the type of vitamin D (native or activated vitamin D). Interestingly, overexpression of FGF23 markedly reverses phosphate-induced vascular smooth muscle cells calcification [29]. As kidney function declines, circulating concentrations of FGF23 rise progressively, most probably as a consequence of rising serum phosphorus concentrations [3, 30]. Higher serum phosphorus concentrations in comparison with healthy controls have also been reported in patients with advanced HF [31]. Excessively enhanced FGF23 concentrations of several hundred and up to several thousand pg/ml have been reported in both patients with end-stage renal disease and patients with end-stage heart failure [32, 33]. Since there is evidence that mortality risk rises gradually and progressively as FGF23 increases [6, 34], the vitamin D-induced mean increase in FGF23 of 300 pg/ml in patients with end-stage organ failure may be predictive for detrimental effects on health. The clinical relevance of our findings, in particular the question of whether the vitamin D-induced FGF23 increase exerts potential adverse effects or is simply a beneficial defense mechanism against high phosphorus levels warrants further investigations.

The vitamin D-induced effects on FGF23 were also influenced by baseline 25(OH)D concentrations, with a significantly higher mean FGF23 increase at 25(OH)D concentrations <50 nmo/l (FGF23: +34 pg/ml) than at concentrations ≥50 nmol/l (FGF23: +9 pg/ml). This can be explained by a vitamin D-induced increase in phosphorus absorption, which may reach a plateau at adequate 25(OH)D concentrations (i.e., ≥50 nmol/l). However, out of the 16 studies with baseline 25(OH)D concentrations <50 nmol/l, eight were performed in CKD patients and it is also possible that results were influenced by study population characteristics, i.e., higher vitamin D-induced increase in FGF23 concentrations in patients with versus without CKD.

The present meta-analysis has the limitation that published studies used assays which measured different forms of FGF23 such as cFGF23 and iFGF23. Although a conversion factor proposed for the comparison of cFGF23 with iFGF23 concentrations [14], was also used in this meta-analysis, some uncertainty regarding the comparison of different FGF23 forms remains. Moreover, publication bias cannot be ruled out. It is noteworthy that funnel plots for assessing publication bias are constructed by plotting the regression of observed effect sizes against the corresponding SEs, weighted by the inverse of the pooled variance, assuming that higher SEs indicate smaller studies. In the present meta-analysis, however, standard deviations, and thus SEs, vary widely, even between studies with similar numbers of patients (Fig. 2). Consequently, the method of assessing publication bias may be questioned in this specific analysis, and publication bias may be lower than assumed by inspection of the funnel plot. In addition, the subgroup analysis of studies with daily vitamin D intakes ≤2000 IU was based on three studies only. Therefore, results have to be confirmed in future analyses with more trials. Finally, there was substantial heterogeneity among studies and this heterogeneity may only partly be explained by the sensitivity analyses we performed. Nevertheless, it is noteworthy that some of the heterogeneity such as the extremely high FGF23 in the study by Ramirez-Sandoval et al. [35] ties well with the extremely low calcitriol levels in that study because FGF23 inhibits calcitriol synthesis.

In summary, this meta-analysis of RCTs shows that vitamin D administration increases concentrations of the cardiovascular risk marker FGF23. However, subgroup analyses indicate that relatively low daily doses do not influence FGF23 substantially, whereas the effect is most pronounced in patients with end-stage kidney/heart failure, and if activated vitamin D is given.

## Supplementary information

Supplementary Information
