# Correction: The effect of vitamin D on fibroblast growth factor 23: a systematic review and meta-analysis of randomized controlled trials

**DOI:** 10.1038/s41430-021-01019-9

**Published:** 2021-10-12

**Authors:** Armin Zittermann, Heiner K. Berthold, Stefan Pilz

**Affiliations:** 1 Clinic for Thoracic and Cardiovascular Surgery, Herz- und Diabeteszentrum NRW, Ruhr University Bochum, Bad Oeynhausen, 32545 Germany; 2 Department of Internal Medicine and Geriatrics, Bethel Clinic (EvKB), Bielefeld, 33611 Germany; 3 Division of Endocrinology and Diabetology, Department of Internal Medicine, Medical University of Graz, Graz, 8036 Austria

**Keywords:** Biomarkers, Endocrine system and metabolic diseases, Cardiovascular diseases

Correction to: *European Journal of Clinical Nutrition* 10.1038/s41430-020-00725-0, published online 27 August 2020

The article “The effect of vitamin D on fibroblast growth factor 23: a systematic review and meta-analysis of randomized controlled trials”, written by Armin Zittermann, Heiner K. Berthold, Stefan Pilz was originally published Online First without Open Access. After publication in volume 75, issue 6, page 980–987, the author decided to opt for Open Choice and to make the article an Open Access publication. Therefore, the copyright of the article has been changed to © The Author(s) 2020 and the article is forthwith distributed under the terms of the Creative Commons Attribution 4.0 International License, which permits use, sharing, adaptation, distribution and reproduction in any medium or format, as long as you give appropriate credit to the original author(s) and the source, provide a link to the Creative Commons licence, and indicate if changes were made.

The images or other third party material in this article are included in the article’s Creative Commons licence, unless indicated otherwise in a credit line to the material. If material is not included in the article’s Creative Commons licence and your intended use is not permitted by statutory regulation or exceeds the permitted use, you will need to obtain permission directly from the copyright holder.

To view a copy of this licence, visit http://creativecommons.org/licenses/by/4.0/.

Funding Open Access funding enabled and organized by Projekt DEAL.

