# Supplementary material for: The effect of vitamin D on fibroblast growth factor 23: a systematic review and meta-analysis of randomized controlled trials
Source: Eur J Clin Nutr. 2020 Aug 27;75(6):980–7. doi: 10.1038/s41430-020-00725-0 (PMC8510890; doi:10.1038/s41430-020-00725-0)

Supplementary Information

The effect of vitamin D on fibroblast growth factor 23: A systematic review and meta-analysis of randomized controlled trials

Zittermann A, Berthold HK, Pilz S

		Page
**Supplemental Table 1:**	List of included studies	2
**Supplemental Table 2:**	Initial and achieved circulating 25-hydroxyvitamin D concentrations in studies using native vitamin D or 25-hydroxyvitamin D supplements	3
**Supplemental Figure 1:**	Funnel plot of included studies	4
**Supplemental Figure 2:**	Methodological quality graph: Authors´ judgements about each methodological quality item presented as percentages for included studies	5

Supplemental Table 1: List of included studies

Article	Study	Author	Year	Journal	Region	Mean age years	Vit. D type	Vitamin D dose	Vit. D dose equivalent per day	Assay	Study duration weeks	Mean Initial 25OHD nmol/l	Health status
1	1	Burnett-Bowie	2012	Clin J Am Soc Nephrol	Amer	27	D2	50,000 IU/wk	7143 IU/d	Kainos	12	45	Healthy
2	2	Carpenter	2014	JCEM	Amer	41	paricalcitol	1-3 µg/d	1-3 µg/d	Kainos	52	70	XLH
3	3	Carvalho	2017	PlosOne	Amer	59	D3	100,000 IU/wk	14,286 IU/d	R&D	12	40	CKD5
4	4	Cheng	2018	Clin Interv Aging	Asia	58	calcitriol	0.5 µg/d	0.5 µg/d	Kainos	12	43	postm.
5	5	De Boer	2013	Kidney Int	Amer	66	paricalcitol	2 µg/d	2 µg/d	Kainos	8	29	CKD3-4
6	6	Gravesen	2013	Scand J Clin Lab Invest	Eur	>18	D2	50,000 IU/wk	7143 IU/d	Immutopics	6	63	CKD4-5
7	7	Havens	2014	Antivir Ther	Amer	22	D3	50,000 IU/mo	1786 IU/d	Kainos	12	n.s.	HIV-inf.
8	8	Kamelian	2018	J Endocrinol Invest	Asia	40	D3	50,000 IU/wk	7143 IU/d	Biosystems	12	21	n.s.
9	9	Lerch	2018	Nephrol Dial Transplant	Eur	9	D2	2000 IU/d	2,000 IU/d	Immutopics	52	50	CKD2-5
10	10	Levin	2017	CJASN	Amer	66	calcitriol	0.5 µg trice wk	0.21 µg/d	Kainos	26	67	CKD3-4
10	11	Levin	2017	CJASN	Amer	66	25OHD	5000 IU trice wk	2143 IU/d	Kainos	26	65	CKD3-4
11	12	Macdonald	2013	JBMR	Eur	65	D3	400 IU/d	400 IU/d	Kainos	52	33	postm.
11	13	Macdonald	2013	JBMR	Eur	65	D3	1,000 IU/d	1,000 IU/d	Kainos	52	33	postm.
12	14	Marckmann	2012	Nephrol Dial Transplant	Eur	70	D3	40,000 IU/wk	5714 IU/d	Kainos	8	27	CKD 4-5
13	15	Mesinovic	2019	J Steroid Biochem Mol Biol	Aus	30	D3	4,000 IU/d	4,000 IU/d	Immutopics	16	31	obese
14	16	Nygaard	2014	Plos One	Eur	45	D3	3000 IU/d	3000 IU/d	Kainos	16	32	healthy
15	17	Ramirez-San.	2019	J Nephrol	Amer	41	D3	4800 IU/d	4,800 IU/d	Magpix Sys.	16	29	CKD 5
16	18	Seibert	2013	Nephron Clin Pract	Eur	67	D3	20,000 IU/mo to 40,000 IU/wk	658-5714 IU/d	Immutopics	12	29	CKD5
17	19	Spoto	2018	Nephrol Dial Transplant	Eur	62	paricalcitol	2 µg/d	2 µg/d	Kainos	12	33	CKD3-4
18	20	Trummer	2018	Eur J Nutr	Eur	60	D3	2000 IU/d	2000 IU/d	Biomedica	8	55	Hypert.
19	21	Westerberg	2018	Nephrol Dial Transplant	Eur	63	D3	8000 IU/d	8000 IU/d	Kainos	12	57	CKD3-4
20	22	Yadav	2018	J Bone Miner Res	Asia	44	D3	300,000 IU bimo	5357 IU/d	Immutopics	16	33	CKD 3-4
21	23	Zittermann	2018	Int J Endocrinol	Eur	53	D3	4000 IU/d	4000 IU/d	Immutopics	156	36	HF D

Abbreviations: Eur, Europe; Amer, America, Aus, Australia; n.s., not specified; d, day; wk, week; mo, month; bimo, bimonthly; XLH, x-linked hypophosphatemia; CKD, chronic kidney disease; HIV-inf., human immunodeficiency virus infected; postm., postmenopausal; hypert., hypertension; HF D, heart failure stage D; 25OHD, 25-hydroxyvitamin D

Supplemental Table 2: Initial and achieved circulating 25-hydroxyvitamin D concentrations in studies using native vitamin D or 25-hydroxyvitamin D supplements

Author	Vit. D type	Vitamin D dose	Mean Initial 25(OH)D	Mean Achieved 25(OH)D	Mean Treatment effect FGF23	Health status
Burnett-Bowie, 2012	D2	50,000 IU/wk	45	107	30	Healthy
Carvalho, 2017	D3	100,000 IU/wk	40	107	923	CKD5
Gravesen, 2013	D2	50,000 IU/wk	63	129	9	CKD4-5
Havens, 2014	D3	50,000 IU/mo	n.s.	80	4	HIV-inf.
Kamelian, 2018	D3	50,000 IU/wk	21	90	3	n.s.
Lerch, 2018	D2	2000 IU/d	50	82	3	CKD2-5
Levin, 2017	25OHD	5000 IU trice wk	65	147	24	CKD3-4
Macdonald, 2013	D3	400 IU/d	33	65	-9	postm.
Macdonald, 2013	D3	1,000 IU/d	33	76	-7	postm.
Marckmann, 2012	D3	40,000 IU/wk	27	155	402	CKD 4-5
Mesinovic, 2019	D3	4,000 IU/d	31	88	9	obese
Nygaard, 2014	D3	3000 IU/d	32	88	2	healthy
Ramirez-San., 2019	D3	4800 IU/d	29	100	8075	CKD 5
Seibert, 2013	D3	20,000 IU/mo to 40,000 IU/wk	29	88	-202	CKD5
Trummer, 2018	D3	2000 IU/d	55	90	1	Hypert.
Westerberg, 2018	D3	8000 IU/d	57	102	4	CKD3-4
Yadav, 2018	D3	300,000 IU bimo	33	95	-8	CKD 3-4
Zittermann, 2018	D3	4000 IU/d	36	100	340	HF D

Abbreviations: n.s., not specified; d, day; wk, week; mo, month; bimo, bimonthly; XLH, x-linked hypophosphatemia; CKD, chronic kidney disease; HIV-inf., human immunodeficiency virus infected; postm., postmenopausal; hypert., hypertension; HF D, heart failure stage D

Supplemental Figure 1: Funnel plot of included studies

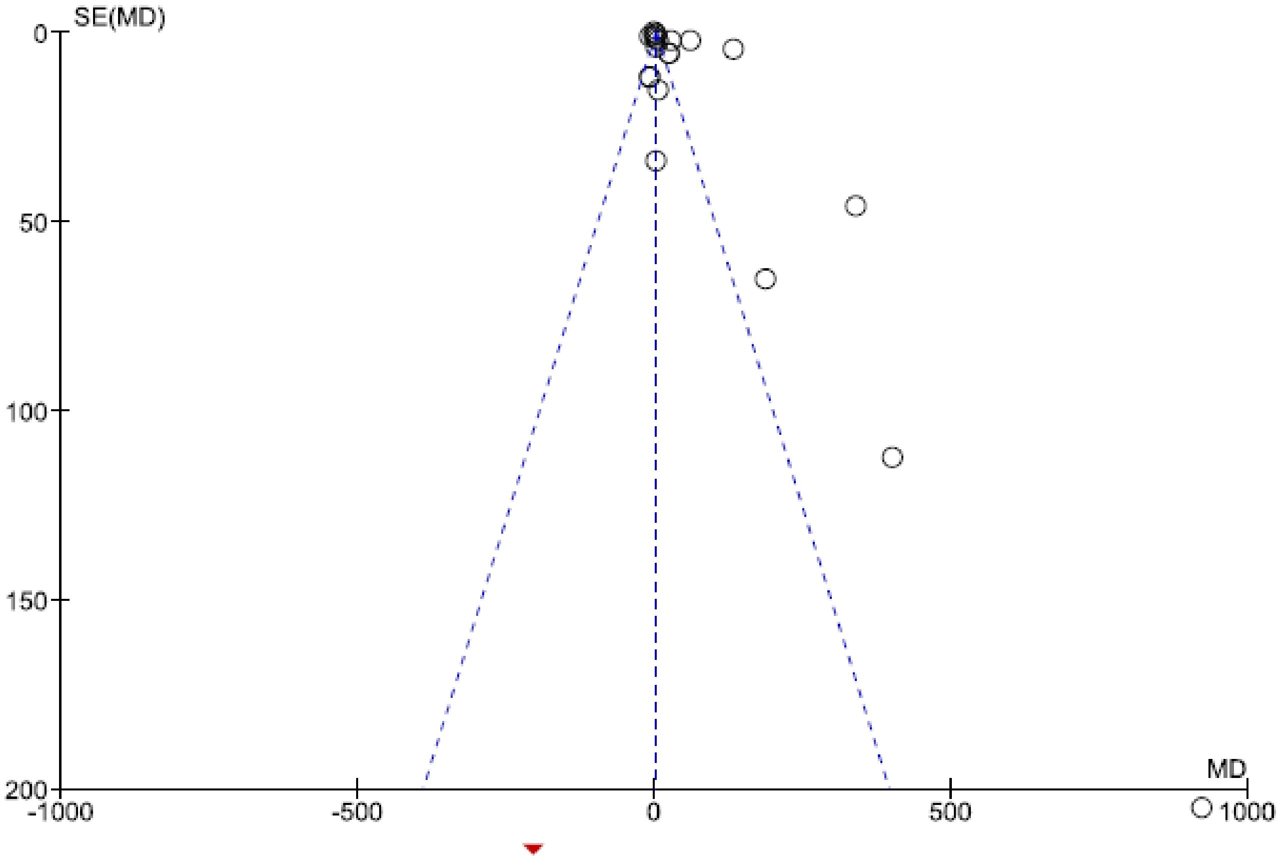

Figure legend: MD indicates the mean difference in FGF23 concentration between the vitamin D and placebo group in individual trials; each circle displays the result of an individual study; a negative MD notifies a decrease in FGF23 by vitamin D supplementation; SE (MD) denotes the standard error of the mean difference. Risk of publication bias cannot be ruled out if circles are lying outside the dotted lines.

Supplemental Figure 2: Methodological quality graph: Authors´ judgements about each methodological quality item presented as percentages for included studies

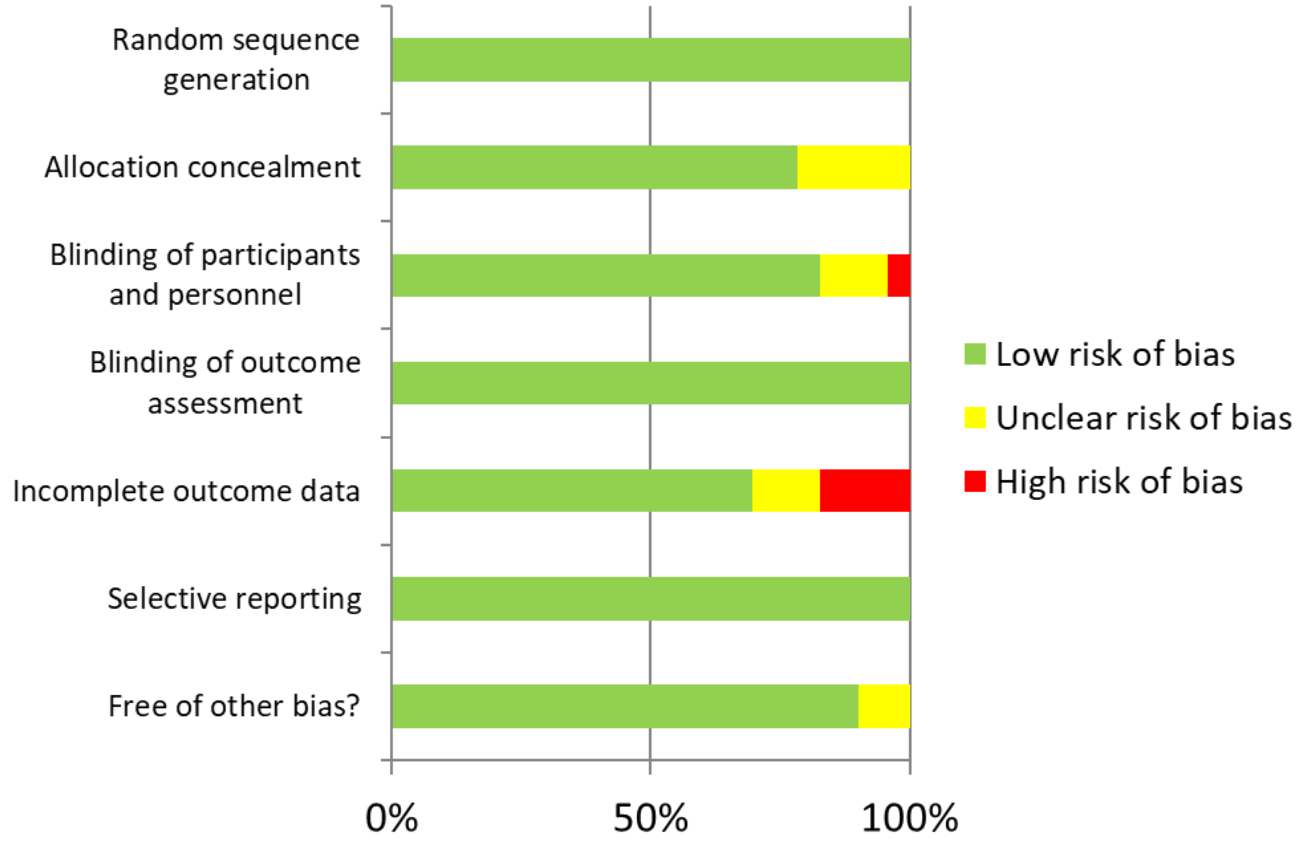

Supplement: Supplementary file 1 — Supplementary Information [file 41430_2020_725_MOESM1_ESM.pdf]
